# C1-C2 Dorsal Epidural Hematoma on Cervical Spine CT: A Novel Imaging Sign of Posterior Atlanto-Occipital Membrane Stripping Injury

**DOI:** 10.7759/cureus.19543

**Published:** 2021-11-13

**Authors:** Peter Fiester, Erik Soule, Gazanfar Rahmathulla, Dinesh Rao

**Affiliations:** 1 Neuroradiology, University of Florida College of Medicine, Jacksonville, USA; 2 Interventional Radiology, University of Florida College of Medicine, Jacksonville, USA; 3 Neurological Surgery, University of Florida College of Medicine, Jacksonville, USA

**Keywords:** trauma, magnetic resonance imaging, computed tomography, posterior atlanto-occipital membrane, dorsal epidural hematoma

## Abstract

Background and Purpose: Identify patients with a dorsal epidural hematoma at C1-C2 and examine the major craniocervical junction ligaments for injury on follow-up magnetic resonance imaging.

Materials and Methods: Adult and pediatric trauma patients who suffered a dorsal epidural hematoma at C1-C2 were identified using Nuance mPower software (Nuance Communications, United States). The cervical computed tomography and magnetic resonance imaging exams for these patients were reviewed for craniocervical junction osteoligamentous injuries. An age-matched control group was obtained.

Results: Eight trauma patients were identified with a dorsal epidural fluid collection at C1-C2. All patients with a dorsal epidural hematoma, who underwent follow-up cervical magnetic resonance imaging demonstrated a stripping injury of the posterior atlanto-occipital membrane from the C1 posterior arch with increased short tau inversion recovery signal in the posterior atlanto-occipital membrane complex. Disruption of additional major craniocervcial ligaments on magnetic resonance imaging was relatively common with the most frequently associated ligamentous injuries involving the tectorial membrane (five patients) followed by the alar ligaments and anterior altanto-occiptial membrane (four patients each).

Conclusions: A C1-C2 dorsal epidural hematoma is a rare injury that may be identified on cervical spine computed tomography but may be easily overlooked by the radiologist. We propose that a C1-C2 dorsal epidural hematoma is a direct result of a significant hyperflexion-hyperextension force with subsequent stripping of the posterior atlanto-occipital membrane from the posterior C1 arch. Trauma patients with a C1-C2 dorsal epidural hematoma on cervical spine computed tomography should undergo a cervical magnetic resonance imaging examination to evaluate the integrity of the posterior atlanto-occipital membrane complex and remaining craniocervical junction ligaments for injury.

## Introduction

A dorsal epidural hematoma (DEH) at C1-C2 is a hemorrhage confined between the posterior atlanto-occipital membrane complex (PAOMc) and the posterior dura mater. The proposed mechanism for a post-traumatic DEH at C1-C2 is related to a shearing injury of the dorsal venous plexus within the posterior epidural space. The PAOMc consists of the posterior atlanto-occipital membrane (PAOM), which extends between the occiput and posterior C1 arch, and the posterior atlantoaxial membrane (PAAM), which extends between the posterior C1 arch and C2 lamina [1]. The anterior boundary of the PAOMc is attached directly to the posterior spinal dura at C1, whereas the posterior boundary forms a myoligamentous complex with the rectus posterior major and minor muscles tendons and fibers of the ligametum nuchae [2-3]. The exact origin of the ligamentous fibers that comprise the PAOMc is debated, but recent cadaveric examinations suggest that PAOMc fibers primarily arise from the rectus capitis posterior minor fascia and a vascular sheath surrounding the bilateral vertebral arteries [4,5].

The contribution of the PAOMc toward maintaining craniocervcial junction (CCJ) integrity remains controversial. Whereas some prior researchers suggest that the PAOMc is a major CCJ ligament and primary stabilizer between the occiput and upper cervical spine, more recent studies suggest it plays a nominal role in CCJ integrity when compared to the contribution of the alar ligaments, atlanto-occipital joint/capsular ligaments, tectorial membrane, and transverse band of the cruciform ligament [6-8].

On cervical magnetic resonance imaging (MRI), the PAOMc is easily identified as a dark, midline T2 hypointense band of variable thickness, which is indistinguishable anteriorly from the posterior spinal dura at C1 and C2. Small, fan-shaped T2 hypointense fibers of the PAOMc are often identified paramidline on T2 weighted sequences between the occiput and posterior C1 arch, where contributions from the ligamentum nuchae and rectus capitis posterior muscle tendons are present. In the setting of a significant hyperflexion-hyperextension force (whiplash injury) directed at the CCJ, increased short tau inversion recovery (STIR) signal in the PAOMc may occur, suggesting the presence of fluid or edema within the PAOM and PAAM [9].

A C1-C2 DEH is a rare imaging finding, and to our knowledge, the medical literature does not provide an examination of the potential mechanism for a hematoma in this specialized compartment in the acute trauma setting. Thus, a retrospective analysis of the imaging data from patients with a C1-C2 DEH on cervical spine computed tomography (CT) was undertaken to better define the imaging features of a DEH confined to C1-C2 in the trauma population, including the size and extent of the hematoma. Given the anatomic relationship between the PAOMc, posterior C1-C2 dura mater, and posterior epidural venous plexus, the craniocervical ligaments were closely examined in patients who subsequently underwent cervical MRI. Finally, the clinical presentation, management, and global outcome of our patient population were analyzed. 

## Materials and methods

A waiver of informed consent was granted to retrospectively evaluate the imaging and clinical findings of trauma patients, who suffered a dorsal epidural hematoma at C1-C2 on cervical spine CT exams. All patients with a non-contrast-enhanced cervical spine CT exam generated from the emergency department between January 2015 and January 2021 (26,251 patients) were included in the study. Nuance mPower software (Nuance Communications, United States) was utilized to perform a keyword search of the cervical CT radiology reports using the keywords ‘posterior epidural hematoma’ and ‘dorsal epidural hematoma’ to identify patients with a dorsal epidural hematoma at C1-C2. Patients with epidural hematomas in the ventral epidural space or inferior to the C2 level were excluded from the study. CT and MRI exams were performed using the standard departmental protocols. CT images were generated with 0.625 mm slice thickness and reconstructed using multiplanar bone and soft tissue algorithms (GE medical systems, [Chicago, Illinois]). MRI studies were performed on a 1.5 Tesla magnet with a head and neck coil (Siemens Avanto [Munich, Germany]). Slice thickness was 3 mm and sagittal T1, T2, STIR, as well as axial T2, and T2 Multi-Echo Data Image Combination (MEDIC) sequences were obtained. All CT and MRI exams were reviewed by two experienced board-certified neuroradiologists with an additional certificate of additional qualification (CAQ) awarded by the American Board of Radiology.

**Figure 1:**
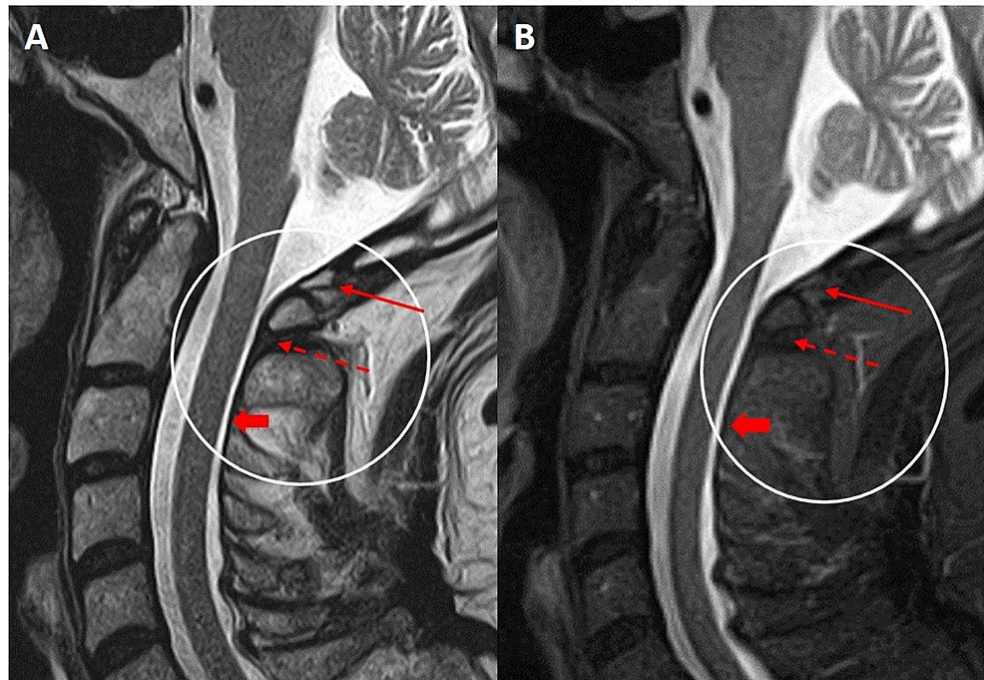
Sagittal T2 weighted (A) and STIR (B) Cervical MRI sequences demonstrating the normal imaging appearance of the posterior atlanto-occipital membrane (red arrow) extending between the opisthion of the occiput and posterior C1 arch. A small area of ossification is present within the membrane. The posterior atlanto-axial membrane (red dashed arrow) extends between the posterior C1 arch and C2 lamina where it is continuous with the ligamentum flavum inferior to C2 (red block arrow). The two membranes are normally indistinguishable from the posterior dura at C1-C2 and together comprise the posterior atlanto-occipital membrane complex. STIR: short tau inversion recovery, MRI: magnetic resonance imaging

A DEH was considered present on CT exams if a hyperdense or isodense hematoma was interposed between the posterior dura mater at C1-C2 and the posterior C1-C2 lamina. Careful attention was made on CT and MRI exams to evaluate for C1-C2 fractures (including microfracture on MRI) and/or epidural blood products which extended inferior to the C1-C2 level. The size of the DEH was measured at its greatest anteroposterior dimension perpendicular to the posterior C1 lamina and the craniocaudal extent was measured parallel to the posterior dura mater. If a cervical MRI exam was performed within 48 hours, the craniocervical junction ligaments were directly inspected for injury. MRI image quality was graded as either poor, intermediate, or diagnostic quality. The posterior atlanto-axial membrane complex was considered injured if there was increased STIR signal in the ligament with a fluid filled space (hematoma) interposed between the PAOM and the posterior C1 arch. A thorough analysis of the integrity of the anterior atlantooccipital membrane, alar ligaments, and transverse ligament of C2 was obtained (Figure 1). 

Evidence of skull base or cervical spine fracture, as well as traumatic brain or cervical cord injury, was recorded. Pertinent demographic information, mechanism of injury, clinical management, and outcomes were recorded. A good clinical outcome/full recovery was defined as the absence of persistent neurologic deficits on follow-up evaluation by the neurosurgery department at least four months following the initial injury.

The total number of non-contrast CT scans of the cervical spine performed in the emergency department between January 2015 and January 2021 was used as a denominator to estimate incidence. Age-matched patients with a history of trauma and MRI confirmed craniocervical junction ligamentous injury, without dorsal epidural hematoma confined to C1-C2 were included as a control group. None of the initial cervical spine CT exams of these patients demonstrated a dorsal epidural hematoma. Of note, one patient from the dorsal epidural hematoma positive cohort did not undergo follow-up MRI. The presence of pAOMc ligamentous stripping injury could not be confirmed, and for the purpose of statistical analysis, this patient was treated as negative for pAOMc stripping injury but positive for dorsal epidural hematoma. The qualitative data was tabulated. Fisher’s exact test was applied to the tabulated qualitative data. The null hypothesis was taken as “there is no relationship between the presence of a dorsal epidural hematoma confined to C1-C2 and stripping injury to the pAOMc” MedCalc software version 19.2.6 (Ostend, Belgium) was used to perform statistical analysis.

## Results

A total of eight patients (four male and four female), including two pediatric patients less than 17 years old, were identified with a DEH at C1-C2 on cervical spine CT (Table 1). 

**Table 1:** Eight patients with a dorsal epidural hematoma confined to the C1-C2 dorsal epidural space on cervical spine CT. Seven of eight patients subsequently underwent follow-up cervical MRI, demonstrating a stripping injury of the posterior altanto-occipital membrane from the posterior C1 arch. CT: computed tomography, MRI: magnetic resonance imaging, MVA: motor vehicle accident, GCS: glasgow coma scale, AAOM: anterior atlanto-occipital membrane, TM: tectorial membrane, SAH: subarachnoid hemorrhage, SH: subdural hematoma, IVH: intraventricular hemorrhage, LUE: left upper extremity, MJC: miami J collar

Sex/Age	Mechanism of Injury	Clinical Signs/Symptoms	Diagnostic Modality	C1-C2 DEH size (AP/CC Dimension - mm)	PAOMC Stripping Injury	Additional CCJ Injuries	Cervical Cord contusion	Intracranial Trauma	Treatment	Outcome
F/ 9	MVA	GCS score, 15	CT/MR	20 x 2.5	yes	AAOM, L alar, TM	no	no	External halo	Full recovery
F/ 40	MVA	GCS score, 6	CT/MR	31 x 5	yes	AAOM, B/l alar, TM	no	SAH, Contusion	External halo	Deceased
M/ 34	MVA	GCS score, 14	CT/MR	25 x 2	yes	AAOM, TM	no	no	MJC	Quadriplegic
M/ 66	MVA	GCS score, 3	CT/MR	25 x 3	yes	L alar	no	SAH, contusion, SH	External halo	LUE weakness
F/ 53	pedestrian vs. automobile	GCS score, 3	CT/MR	21 x 2	yes	AAOM, TM L alar	no	SAH, IVH, contusion, SH	External Halo	LUE weakness
F/ 62	MVA	GCS score, 13	CT/MR	12 x 4	yes	TM	no	no	External Halo	Bilateral upper extremity weakness
M/ 16	MVA	GCS score, 3	CT/MR	21 x 2	yes	none	yes (C2-C3)	SAH, IVH, contusion	Surgical fusion	Lower extremity weakness
M/ 45	MVA	GCS score, 14	CT	24 x 3	MRI not performed	MRI not performed	MRI not performed	no	MJC	Full recovery

All injuries involved a motor vehicle accident, either primary motor vehicle accidents or pedestrian struck by motor vehicles (one patient). Clinical management was variable depending on additional CCJ injuries. Two patients were treated conservatively with Miami J Collar placement, five patients underwent external halo placement, and one patient underwent an occipital cervical fusion. Two patients achieved good functional outcomes without persistent neurologic deficits upon a four-month follow-up exam, five patients exhibited persistent neurologic deficits (weakness, spasticity, quadriplegia: one patient), and one patient passed away.

**Figure 2:**
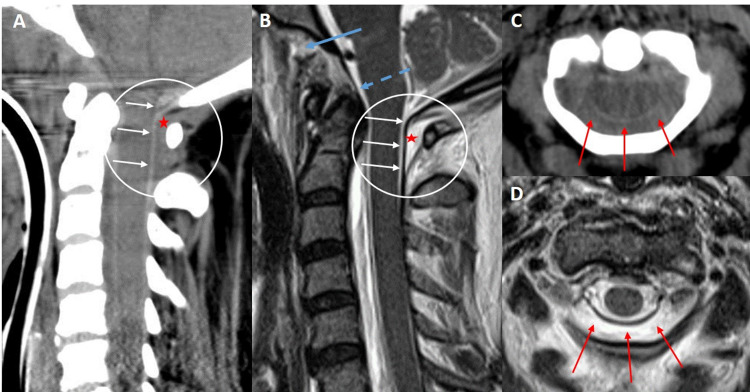
40-year-old female status post motor vehicle accident presenting for cervical CT and MRI exam. Sagittal CT (A) and T2 weighted MRI (B) of the craniocervical junction demonstrate a stripping injury of the posterior dura and posterior atlanto-occipital membrane from the posterior C1 arch (white arrows) with a localized posterior epidural hematoma at C1-C2 (red stars). Associated tears of the anterior atlanto-axial membrane (blue arrow) and a stretch injury of the tectorial membrane (blue dashed arrow) are also present. Axial CT (C) and T2 weighted MRI (D) images of the same patient demonstrate the posterior epidural hematoma between the posterior dura and posterior C1 arch. CT: computed tomography, MRI: magnetic resonance imaging

**Figure 3:**
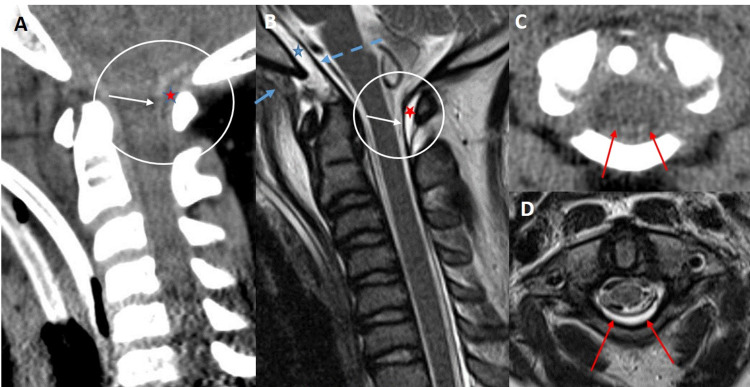
9-year-old female status post motor vehicle accident presenting for cervical CT and MRI exam. Sagittal CT (A) and T2 weighted MRI (B) of the craniocervical junction demonstrate a stripping injury of the posterior atlanto-occipital membrane from the posterior C1 arch (white arrow) with a localized posterior epidural hematoma at C1-C2 (red star). Associated stripping injury of the tectorial membrane (blue dashed arrow) from the posterior clivus with a retroclival epidural hematoma (blue star) with posterior subluxation of C1-C2 in relation to the skull base.  Partial tear of the anterior atlanto-occipital membrane (short blue arrow) is also present. Axial CT (C) and T2 weighted MRI (D) images demonstrate the posterior epidural hematoma localized between the posterior dura and posterior C1 arch (red arrows). CT: computed tomography, MRI: magnetic resonance imaging

The DEH confined to C1-C2 demonstrated an average craniocaudal dimension of 24 mm and median of 23 mm and ranged in size between 12 mm and 31 mm. The average and median anteroposterior dimension of the DEH was 3 mm, respectively, and ranged in size from 2 mm to 5 mm. Average Hounsfield units (HU) averaged 43 with a range between 31 and 52 HU. Grossly, all patients with a DEH did not demonstrate the extension of the epidural hematoma inferior to the C2 lamina (Figures 2-3). Seven patients underwent a follow-up cervical MRI within 48 hours, and all MRI exams were graded as ‘diagnostic’ in quality. In all patients who underwent MRI, there was a stripping injury of the PAOMc from the posterior C1 arch with increased PAOMc STIR signal. A p-value of 0.0014 was obtained. The null hypothesis was rejected, suggesting a correlation between the presence of a DEH confined to C1-C2 and stripping injury to the pAOMc. The incidence of dorsal epidural hematoma in patients who underwent non-contrast cervical spine CT in the emergency department in our level 1 trauma center was calculated as 0.03 percent.

Disruption of additional major craniocervcial ligaments on MRI was relatively common with the most frequently associated ligamentous injuries involving the tectorial membrane (five patients) followed by the alar ligaments, anterior altanto-occiptial membrane, and apical ligament (four patients each). Concomitant skull base and C1-C2 fractures were observed in one patient. One patient suffered a cord contusion, and four patients suffered a traumatic brain injury (hemorrhagic contusion and/or subdural or subarachnoid hemorrhage). 

## Discussion

We present a cohort of eight patients presenting with a localized DEH at C1-C2 on cervical spine CT in the setting of acute trauma with significant whiplash injury. Based on the follow-up cervical MRI findings and the insertion of the PAOMc on the posterior spinal dura at C1-C2, we propose that a DEH confined to this location is the direct result of a stripping injury of the PAOMc from the posterior C1 arch with resultant shearing injury of the dorsal epidural venous plexus. Moreover, the presence of C1-C2 DEH on cervical spine CT was associated with major CCJ ligamentous injury in over half of patients with six patients requiring either prolonged external halo placement or operative fusion for craniocervical disassociation spectrum injury and instability. Associated traumatic brain injury was present in half of the patients, as was persistent neurologic deficits on the follow-up exam.

Although a rare imaging finding, recognition of C1-C2 DEH on cervical spine CT as a sign of significant CCJ injury is vitally important (Figure 2). 

First, several studies have demonstrated a direct association between a delay in CCJ injury diagnosis and spinal stabilization with increasing morbidity and mortality [10,11]. The delay between prompt recognition of CCJ instability may in part account for the high mortality rates (>90%) associated with these injuries. Second, several normative skull base lines and relationships, including the occipital condylar-C1 joint space (condylar-C1 interval), are used to predict CCJ injury on cervical spine CT [12-14]. However, with the increasing availability of cervical MRI, including the utilization of high-resolution, multiplanar imaging, to directly evaluate the CCJ ligaments for injury, recent research studies have suggested that potentially unstable CCJ osteoligamentous injuries may occur in the absence of condylar-C1 widening or an abnormality in the other skull base lines [15,16]. Thus, recognition of the importance of a C1-C2 DEH on cervical CT provides key imaging finding to indicate major CCJ injury and guide patient care toward prompt neurosurgical evaluation and follow-up cervical MRI.

Interestingly, in all seven patients with a stripping injury of the PAOMc confirmed on cervical MRI, the resultant DEH never extended inferior to the C2 lamina. This may be related to the PAOMc being more firmly adherent to the C2 lamina compared to the posterior C1 arch (Figure 3). 

Anatomically, the PAOMc consists of two named components: 1) PAOM: extending between the occiput and C1 and 2) PAAM: extending between C1 and C2. Cadaveric studies performed by Tubbs et al. have demonstrated that the PAAM is continuous with the ligamentum flavum inferior to the C2 level [3,8]. Cervical MRI features of our patient cohort suggest that the PAOM is the primary component stripped off the posterior C1 arch with the anteroposterior dimension of the DEH greatest at the C1 level. A thin ligamentous structure coursing diagonally from the posterior C1 arch and inserting on the posterior spinal dura at the C2 lamina was present in all patients and formed the posterior boundary of the DEH. This structure may represent the PAAM with fluid (hematoma) interposed between the posterior dura at C1-2 and the PAAM. We propose that this ligamentous structure is likely a component of the PAAM, which confines the DEH, so hematoma does not extend inferior to the junction between the PAAM and ligamentum flavum. 

Additional major CCJ ligamentous injuries were common on follow-up cervical MRI in our patient cohort with C1-C2 DEH. The tectorial membrane (five patients) followed by the alar ligaments (four patients) were the most injured major CCJ ligaments. The TM is a strong ligament that incorporates directly with the retroclival dura mater and acts as the superiorly directed extension of the PLL at the C1-C2 level. Functionally, it serves as a primary CCJ stabilizer that helps limit hyperextension and prevents the dens from impinging on the cervical cord [17]. The alar ligaments are strong, bilateral ligamentous bands extending horizontally or slightly craniocaudally between the inferomedial occipital condyle and the tip of the C2 dens. Functionally, they are a major stabilizing CCJ ligament that tightly holds in place the central skull base with the atlantoaxial joint and reduces axial rotation of the contralateral atlanto-occipital joint [2,18]. The prevalence of concomitant injury to the major TM and alar CCJ ligaments, which both are not evaluated by CT imaging, suggests two major points. First, that a C1-C2 DEH is a sign of a high-velocity hyperflexion-hperextension force directed at the CCJ and, second, that a C1-C2 DEH may be a common imaging finding of significant cranciocervical disassociation spectrum injury and instability. This point is supported by the fact that 75% of our patient cohort required long-term immobilization either with external halo placement or surgical fusion.

Chang et al. investigated the utility of isolated increased STIR signal in the PAOMc on MRI as a secondary finding of C1-C2 fractures with high sensitivity (89.7%) [19]. However, more recent research on PAOMc injury grading on cervical MRI demonstrated only half of the patients with increased STIR signal in the PAOMc had a C1-C2 fracture, and only one patient in our cohort demonstrated a C1-C2 fracture (type 2 dens fracture) [20]. 

We acknowledge the DEH size and location may be challenging to appreciate on cervical spine CT in some trauma patients. For our patient cohort, the average craniocaudad dimension of the DEH measured 2.4 cm, while the average anteroposterior dimension only measured 3 mm. The DEH was also clearly confined to the C1-C2 level. For trauma patients with a relatively small DEH, the intrinsic contrast between the CSF space, the posterior dura mater, and epidural hematoma may not be sufficient in order to visualize the DEH on cervical CT. Motion degradation and streak artifact from the skull base or external devices may also hinder evaluation for epidural hematoma. Utilizing trauma spine protocols with thin section (< 1mm) image acquisition and a soft tissue algorithm may help mitigate the challenge of evaluating for spinal epidural hematoma and should be routinely performed in the trauma setting. Interestingly, neither patient with the smallest C1-C2 DEH (12x4 mm; 21x2 mm) in our cohort demonstrated a major CCJ ligamentous injury raising the possibility that increasing the DEH size may correlate with CCJ ligamentous severity.

Our primary limitation was the small sample size (eight patients). In addition, given the large number of trauma cervical CT exams performed during these five-year period (over 25,000 cervical CT exams), this limited the feasibility of inspecting every cervical CT exam and developing a denominator to perform further statistical analysis and determine true incidence of a C1-C2 DEH. Given the subtlety of this finding, we acknowledge there is a high likelihood we did not capture all patients with a C1-C2 DEH. However, since this imaging finding has not been previously reported, it is likely rare and not present in all patients with CCJ trauma. We hope that by being the first to describe the potential mechanism and CT appearance of C1-C2 DEH in the trauma setting, larger patient cohorts with CT and MRI can be collected and evaluated. Finally, our study was a retrospective review, and the possibility of interpreter error and reinforcement bias is possible. 

## Conclusions

A DEH confined to the C1-C2 epidural space on cervical spine CT in the trauma setting is a novel imaging finding indicative of a significant CCJ injury. Based on the anatomy of the PAOMc and posterior spinal dura at C1-C2, we propose that a C1-C2 DEH is the result of a stripping injury of the PAOM from the posterior C1 arch with an associated shearing injury of the dorsal epidural venous plexus. Trauma patients with a C1-C2 DEH on cervical spine CT should undergo cervical MRI to directly evaluate the integrity of the PAOMc and additional CCJ ligaments.
